# Supplementary material for: Voices from Akplabanya: Community adaptation and social-ecological changes in coastal Ghana
Source: Camb Prism Coast Futur. 2025 Sep 3;3:e19. doi: 10.1017/cft.2025.10011 (PMC12573693; doi:10.1017/cft.2025.10011)
Supplement: Galappaththi et al. supplementary material [file S2754720525100115sup001.docx]

**S01: Topic Guide for Semi-Structured Interviews**

**Q1: How have these people experienced climate change?**

1. History and timeline of the people and the place (e.g., table/figure with event name, description, year, etc.):

- First flood ever experienced and the most recent flood caused by tidal waves or rainfall.
- Cultural changes observed or practiced in Akplabanya.
- Describe, sketch, or point out what exists today and what no longer exists.

1. Reflections on the hometown and changes observed over the years.
2. Evidence of climate change and its impacts on participants' lives.
3. The role of coastal erosion, rain, and tides:

- Beliefs about climate change.
- Perceived changes, effects, and impacts of the climate.
- Identification of the social groups most affected.

**Q2: How have these people experienced social-ecological system changes?**
5. Connections between the people and their environment:

- What does the sea and the fish within it mean to you?
- What does the Akplaba River mean to you?
- What do the livestock in Akplabanya mean to you?
- What does the land of Akplabanya mean to you?
- What does the vegetation in Akplabanya mean to you?

1. Mapping social-ecological system changes:

- What are all the changes in the linkages mentioned above?

1. List the resources and describe how they have changed over the years (e.g., water, land, sea, vegetation, education, internal and external support, traditions).
2. How has the way of life in the community changed over the years? Consider aspects like:

- Food, clothing, hairstyles, and accessories/adornments.
- Can you show photos to illustrate these changes?

1. What are the primary drivers of these changes?
2. What are the secondary drivers of these changes?

**Q3: What are the community perceptions of climate change impacts?**
11. Perceptions about sea-level rise and its implications:

- Do buried buildings provide evidence of this change?
- What is the perceived direction of change?

1. Perceptions of flooding, tidal waves, and storm surges:

- How have occurrences per year changed over time?
- What has been lost during flooding, tidal waves, or storm surges?

1. Perceptions of rainfall changes over the years:

- How have occurrences per year changed?
- What has been lost during periods of heavy rain?

1. Perceptions of the rate of sand deposition on the beach of Akplabanya and its direction of change.
2. Perceptions about future changes in sea levels.
3. Perceptions about future flooding, tidal waves, and storm surges, and their direction of change.

**S02: Topic Guide for Key Informant Interviews**

1. How many years ago was Akplabanya established, and what is the history of the community?
2. When did the first Christian missionaries arrive in Akplabanya, and what is their historical significance?
3. How many years ago was formal education introduced in Akplabanya?
4. Can you share the history of the first canoe ever made in Akplabanya?
5. Can you tell me about the first whale that washed ashore in Akplabanya?
6. What was the first god worshipped in Akplabanya, and how many years ago was that?
7. Can you provide the history of the first vehicle brought to Akplabanya?
8. What is the history of the first Indigenous healing practice in Akplabanya?
9. When was the first clinic established in Akplabanya, and what is its history?
10. When did the first livestock disease outbreak occur in Akplabanya?
11. How many years ago was Premise fuel introduced in Akplabanya, and what is its history?
12. When did the first outboard motor arrive in Akplabanya, and what is its historical significance?
13. How many years ago did commercial fishing begin in Akplabanya, and what is its history?
14. When was the first convenience store established in Akplabanya?
15. When was the first alcoholic bar opened in Akplabanya?
16. When was electricity introduced in Akplabanya?
17. What was the first major disease outbreak in Akplabanya, and what is its history?
18. Who was the last clan to settle in Akplabanya, and how many years ago was that?
19. After electricity was introduced, when did Akplabanya experience its first blackout?
20. Was COVID-19 ever recorded in Akplabanya?
21. When did Akplabanya begin practicing light fishing, and what is the history of this practice?
22. In which year did the government attempt to stop light fishing, and what is the history behind this action?
23. When was the first milling machine brought to Akplabanya, who introduced it, and how many years ago was that?
24. Can you share details about UNICEF’s work in Akplabanya?
25. Are there any other memorable events you would like to share?

**S03: Topic Guide for Focus Group Discussions (FGDs)**

1. **Observed Changes**:

- Derived from the compilation of Semi-Structured Interviews (SSIs).

1. **Level of Agreement**:

- Agreement on observed changes discussed during the FGDs.

1. **Causes/Drivers of Observed Changes**:

- Identification of key causes and drivers influencing the observed changes.

1. **Direction of Change**:

- Discussion of the direction of changes (e.g., increasing, decreasing, fluctuating).

1. **Impacts of Observed Changes**:

- Exploration of how these changes have impacted the community and environment.

1. **Potential Future Scenarios**:

- Discussion of possible future scenarios based on current trends and changes.
